# Effects of the Short-Foot Exercise on Foot Alignment and Muscle Hypertrophy in Flatfoot Individuals: A Meta-Analysis

**DOI:** 10.3390/ijerph191911994

**Published:** 2022-09-22

**Authors:** Ching Huang, Liang-Yu Chen, Yi-Hung Liao, Kunanya Masodsai, Yi-Yuan Lin

**Affiliations:** 1 Department of Physical Therapy, Taoyuan General Hospital, Ministry of Health and Welfare, Taoyuan 33004, Taiwan; 2 Department of Exercise and Health Science, National Taipei University of Nursing and Health Sciences, Taipei 11219, Taiwan; 3 Faculty of Sports Science, Chulalongkorn University, Bangkok 10330, Thailand

**Keywords:** intrinsic foot muscle, exercise, navicular drop, foot posture index

## Abstract

This study aimed to conduct a meta-analysis of randomized controlled trials to examine the effects of the short-foot exercise (SFE) compared to foot orthosis or other types of interventions. Eligibility criteria involved participants with flatfoot engaging in the SFE compared to other forms of intervention or control groups without specific intervention. Relevant studies published before the end of June 2022 were identified from databases. A meta-analysis was performed by calculating the mean differences (MD) and standard MD (SMD) using the random effects model. Six trials with 201 patients (out of 609 records) that met selection criteria were reviewed. Five of the six trials implemented distinct interventions in the control group such as shoe insoles and muscle strengthening exercises, while in the remaining trial, controls received no intervention. The SFE group significantly reduced the navicular drop test (NDT) values (MD: −0.23; 95% confidence interval: −0.45 to −0.02; *p* = 0.04) and the foot posture index (FPI-6) score (MD: −0.67; 95% confidence interval: −0.98 to −0.36; *p* < 0.0001) when compared to the control group. The muscle hypertrophy did not differ significantly between the groups. The SFE may contribute more benefits than other intervention as it affects flatfoot individuals’ foot alignment. Hence, the SFE is recommended as a beneficial dynamic support when facing flatfoot problems.

## 1. Introduction

Flatfoot is a common foot condition affecting 2% to 23% of the adult population [1]. It is characterized by the partial or complete collapse of the medial longitudinal arch with rearfoot eversion and forefoot abduction [2], which is associated with changes in lower extremity kinematics during dynamic activity [3]. Common injuries and disorders in connection with flatfoot include Achilles tendinitis, iliotibial band syndrome, knee pain, and low back pain [4]. Previous research has shown that 77% of adults with flatfoot have back or lower extremity pain [5].

Static support, such as foot orthoses, is a common medical intervention for flatfoot problems. A past study found that 50% of symptomatic flatfoot patients were treated using foot orthoses to elevate the medial longitudinal arch and correct deformities [5]. However, a 2018 systematic review and meta-analysis indicated that the effects of foot orthoses on flatfoot were limited, and their impact on foot kinematics was debatable [6]. In addition to relying on foot orthoses to support the arch, strengthening the foot muscles can provide dynamic support.

Extrinsic and intrinsic foot muscles (IFM) support the medial longitudinal arch in the active subsystem [7]. IFM with origins and insertions located in the foot, such as the abductor hallucis, flexor digitorum brevis, and quadratus plantae, play important roles in directly stabilizing the arch [8]. The short-foot exercise (SFE) is one of the most recognized exercises for strengthening the IFM and involves contracting these muscles to pull the first metatarsophalangeal joint toward the calcaneus and raise the medial longitudinal arch without flexing the toes [7,9]. Previous research revealed that the degree of abductor hallucis activation was significantly higher in subjects performing the SFE compared with the traditional toe curl; the SFE is thus considered to be a useful exercise for strengthening IFM [10]. Nonetheless, since the SFE has only been popular for less than a decade, Haun et al. have pointed out that a lack of research and evidence has increased the uncertainty of its benefits [11]. Therefore, a meta-analysis was conducted to investigate the effects of the SFE on flatfoot individuals compared with foot orthoses and other rehabilitation methods.

## 2. Materials and Methods

### 2.1. Selection Criteria

In this study, all the included literature should meet the following criteria: (1) study design: randomized controlled trial (RCT); (2) population: people with flatfoot; (3) short-foot exercise group (SG): SFE, or SFE plus other treatments; (4) control group (CG): other forms of intervention or control groups without a specific intervention. A study would be excluded if it met any of the following criteria: (1) people with other foot problems (such as ankle instability and plantar fasciitis); (2) studies investigating the effect of other foot exercises in the SG; (3) studies investigating the effect of the SFE in the CG; (4) protocols, observational studies, case reports, case series, topics, or reviews; and (5) studies where individual cohorts were reported in duplicate.

### 2.2. Search Strategy and Study Selection

Relevant studies published before the end of June 2022 were identified from the PubMed, Embase, and Cochrane databases, and other sources such as the ClinicalTrials.gov registry (https://clinicaltrials.gov/ (accessed on 30 June 2022)) were utilized to discover unpublished studies. The following MeSH and free-text terms and Boolean operators were used: (flatfoot OR pronat*) AND (short foot OR intrinsic foot muscle). No language or publication date restrictions were applied. The “similar articles” section in PubMed was used to extend the search scope and all related articles retrieved were reviewed. Additional studies were pointed out through searching the reference lists of relevant papers and consulting known experts in the field. The studies were reviewed by two reviewers independently, and discrepancies were resolved by a third reviewer. The meta-analysis was registered in PROSPERO (CRD42022324707).

### 2.3. Data Extraction

Data items including variables (such as participant and intervention characteristics) and outcomes were independently extracted by two reviewers. The following information from each study was collected: study designs, study population characteristics, inclusion and exclusion criteria, intervention types and dosages, and intervention-related parameters. The observations of the two independent individuals were then compared, and any disagreement was resolved by a third reviewer.

### 2.4. Methodological Quality Appraisal

A revised tool to assess the risk of bias in randomized trials (RoB 2) [12] was used by two reviewers independently to assess the methodological quality of each included study. Each study was given an overall risk of bias judgement (i.e., low risk, some concerns, or high risk) according to the following domains: bias arising from the randomization process, bias due to deviations from the intended interventions, bias due to missing outcome data, bias in measurement of the outcome, and bias in selection of the reported result.

### 2.5. Outcomes

The following outcomes were used to evaluate the effects: (1) foot alignment, including navicular drop and foot posture, and (2) muscle hypertrophy.

### 2.6. Statistical Analysis

Review Manager, Version 5.4 (Cochrane Collaboration, Oxford, UK) was used to perform the meta-analysis. All parameters were continuous variables which were analyzed by mean difference (MD) and standard MD (SMD) with 95% confidence intervals (CIs). A random-effects model with an inverse-variance method was selected to analyze these effect measures. Statistical heterogeneity was quantified by I^2^ test. Statistical significance was set at *p*-value < 0.05 for overall effect.

## 3. Results

### 3.1. Study Selection

**Figure 1:**
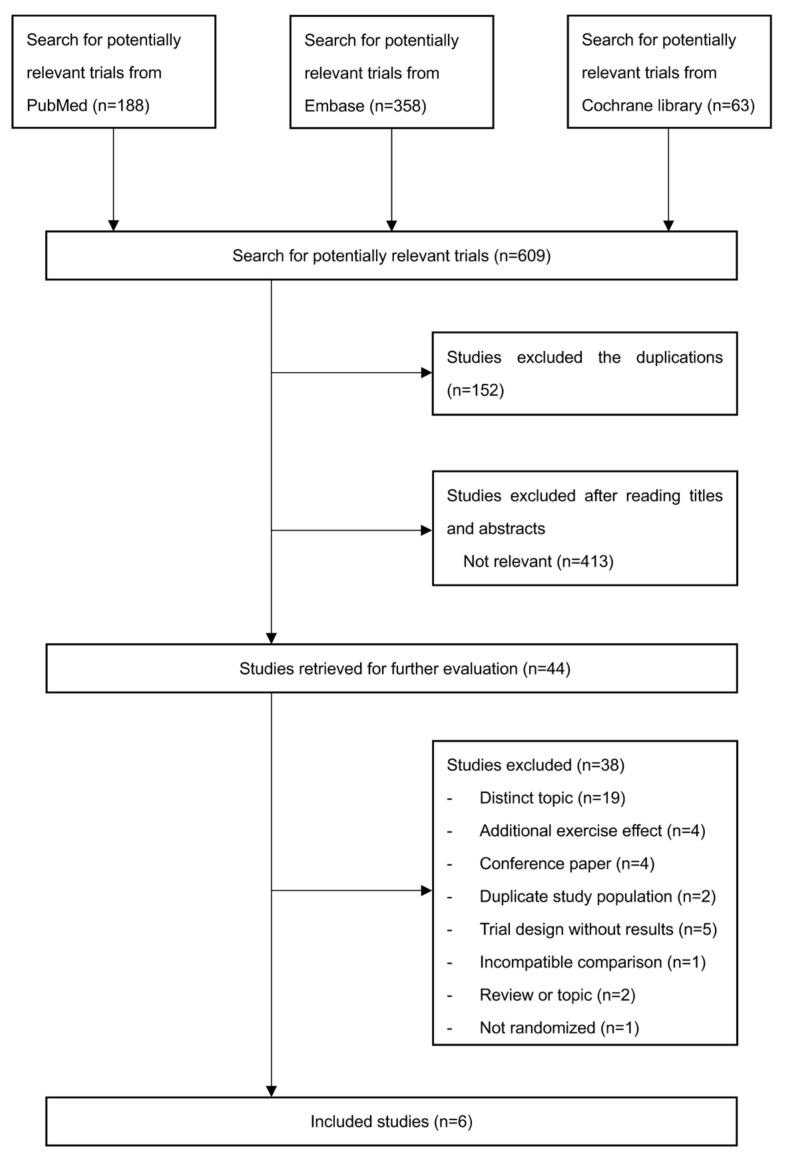
Flowchart of study selection.

The results of the search and selection process are described in the flowchart (Figure 1). Specific search strategies mentioned above yielded 609 studies from different databases, of which 152 and 413 studies were excluded due to duplications or irrelevance after screening titles and abstracts, respectively. Of the 44 retrieved studies, 19 studies were distinct topics, 4 studies included additional ankle or foot exercises (such as the toe curl) in the SG other than the SFE, 4 studies were conference papers with abstracts only, 2 studies were publications with duplicated participants, 5 studies were protocols only, 1 study focused on balance effects, which is different from the outcomes of the current study [13], 1 study was a narrative review [14], 1 study was a critically appraised topic [11], and 1 study was not a RCT [15]. The remaining six studies meeting selection criteria were included in this study [16,17,18,19,20,21].

### 3.2. Study Characteristics

The six RCTs were published between 2011 and 2021 and involved sample sizes ranging from 7 to 43 with a total of 201 participants. The mean age across studies ranged from 19.45 to 43.60 years, where one study contained middle-aged participants [18] and all the others consisted of young adults. The mean BMI across studies ranged from 19.8 to 29.39 kg/m^2^, where one study included only obese people [21] and the rest included adults with a BMI within the healthy weight range (although two studies provided height and weight rather than BMI data). Four studies exclusively performed the SFE in the SG, one study combined foot orthoses [16], and the other study incorporated hip and knee exercises [18] in the SG. The CG in all studies received different kinds of interventions (instead of the SFE) except for a no-treatment CG in a single study [19] (Table 1).

### 3.3. Risk of Bias in Studies

The methodological quality of the included RCTs is summarized in Table 2. No information about concealment of the allocation sequence was detected in four studies [16,17,19,21]. One study had a concealed and random allocation sequence, but baseline differences between groups were found [20]. Participant and/or personnel might have been aware of intervention groups, and unclear information regarding non-protocol interventions was observed in 5 studies [16,17,18,19,21]. Two studies demonstrated a lack of information on outcome assessors being aware of the intervention received by participants [17,18]. One study’s outcome assessors were aware of the intervention received, so the assessment of the outcome could have been influenced by this knowledge, and this was considered to have risks of bias [21]. One study’s outcome data were reported as a median and interquartile range [19].

### 3.4. Navicular Drop

**Figure 2:**
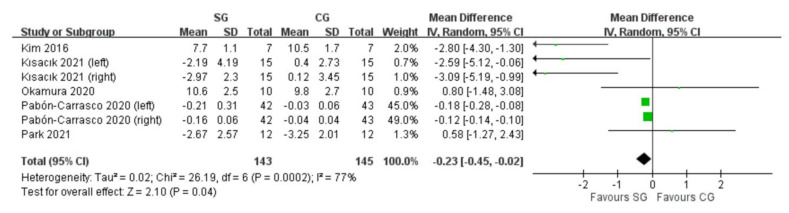
Forest plot of comparison: navicular drop by navicular drop test (NDT); outcome: the SG significantly decreased values of navicular drop compared with the CG. Abbreviations: SG, short-foot exercise group; CG, control group. Green square: point estimate for each study; Black diamond symbol: average effect.

Five RCTs assessed navicular drop by the navicular drop test (NDT) [17,18,19,20,21]. Two RCTs had both feet assessed for each participant [18,20] whereas the others had one tested foot. Change scores were extracted from three RCTs [18,20,21], and post-intervention values were extracted from the others when combining the data. The SG significantly decreased values of navicular drop compared with the CG (MD: −0.23 mm; 95%CI: −0.45 to −0.02). The I^2^ value was 77% which indicated high heterogeneity (Figure 2).

### 3.5. Foot Posture

**Figure 3:**
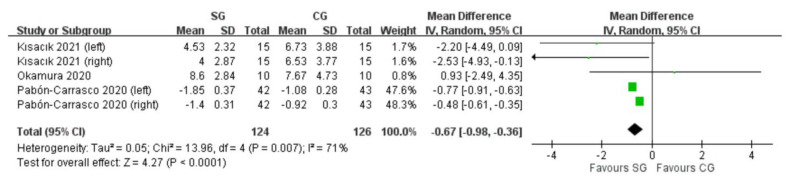
Forest plot of comparison: foot posture by foot posture index (FPI-6); outcome: the SG significantly lowered FPI-6 values compared with the CG. Abbreviations: SG, short-foot exercise group; CG, control group. Green square: point estimate for each study; Black diamond symbol: average effect.

Three RCTs assessed foot posture by the foot posture index (FPI-6) [18,19,20]. Two RCTs had both feet assessed for each participant [18,20] while the other had one tested foot. Change scores were extracted from one RCT [20], and post-intervention values were extracted from the others when combining the data. The means and standard deviations were estimated according to formulas [22] since the RCT’s outcome data were reported as median and interquartile range [19]. The SG significantly lowered FPI-6 values compared with the CG (MD: −0.67 score; 95%CI: −0.98 to −0.36). The I^2^ value was 71%, which indicated moderate heterogeneity (Figure 3).

### 3.6. Muscle Hypertrophy

**Figure 4:**
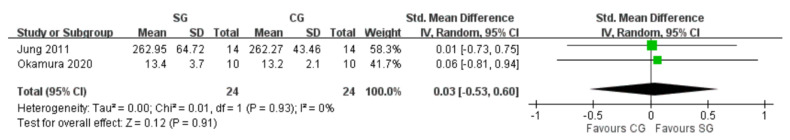
Forest plot of comparison: muscle hypertrophy; outcome: no significant difference was observed between the SG and CG. Abbreviations: SG, short-foot exercise group; CG, control group. Green square: point estimate for each study; Black diamond symbol: average effect.

Two RCTs assessed IFM (abductor hallucis) hypertrophy outcomes; one RCT measured muscle cross-sectional area (mm^2^) [16], and the other measured muscle thickness (mm) [19]. Post-intervention values were extracted from both RCTs. No significant difference was observed between the SG and CG (SMD: 0.03; 95%CI: −0.53 to 0.60). The I^2^ value was 0%, which indicated the absence of heterogeneity across the RCTs (Figure 4).

## 4. Discussion

The meta-analysis indicated that the SFE significantly corrected foot alignment, with the tendency of a decreased navicular drop and a more neutral position compared with the CG. Although there was no difference in muscle hypertrophy between the groups, performing the SFE has evident benefits for flatfoot individuals.

The IFM are the main local stabilizers of the foot as these muscles provide anatomical and biomechanical contributions [7]. The SFE has been described as a practical therapeutic exercise of the IFM in spite of limited evidence. Pabón-Carrasco et al. revealed a trend of more normalized foot alignment through the SFE than through the dorsal and plantar flexion exercise of the metatarsophalangeal joints [20]. While the dorsal and plantar flexion exercise along with the toe curl definitely activate some IFM, they also require considerable activation of the extrinsic foot muscles and are thus less recommended before foot core stability is established [7]. Kim et al. concluded that obvious changes of foot alignment were observed more from applying the SFE than by using foot orthoses [17]. Despite the fact that foot orthoses may increase the afferent feedback, which may result in decreased eversion by contracting inverting muscles [23], the mechanism lacks further foot strengthening references. The above interpretations are supported by the results of our meta-analysis which pointed out that the SFE is more effective than other interventions on normalizing foot alignment.

A previous study with neutral foot alignment subjects reported that the muscle activity of abductor hallucis (one of the IFM) was significantly greater in the SFE group than in the toe curl group [10]. Another trial which is included in the current study stated that additional SFE added extra flexor hallucis (another one of IFM) strength when compared with the original intervention [16]. Since the IFM strength amplitude could be interfered with by superficial tissues perturbation, muscle hypertrophy measurement is an appropriate substitute because a general positive relation exists between muscle strength and muscle hypertrophy. However, our meta-analysis demonstrated a nonsignificant difference of muscle hypertrophy between the SFE and the control group. Jung et al. showed that there was no significant difference in post-intervention values of the cross-sectional area of abductor hallucis between the groups, but the change scores in the cross-sectional area of abductor hallucis were significantly different between the groups; this indicated that subtly different pre-intervention values might lead to alternative interpretations of the results [16]. Okamura et al. revealed a nonsignificant difference in post-intervention values of the abductor hallucis thickness due to the fact that the ultrasound may be insufficient to perceive slight changes in IFM [19]. Although the use of change scores could increase precision [24], it is difficult to obtain all the change score data from each trial. Therefore, more studies are required to confirm the difference of muscle hypertrophy between groups.

Variations in the intervention period may cause diverse results. There is no unified protocol about SFE training as the period of implementation was varied between each scientific study to date. It is critical to train for at least 8 weeks because motor learning, coordination, and the ability of muscle recruitment progress considerably over this time period [25]. Some of the studies found that SFE training was effective after 4 to 6 weeks [17,18,20], but the effect does not appear to increase with the intervention period. As research is currently unable to confirm the effects of different intervention periods owing to the fact that varied training programs, verbal instructions, correction techniques, and training frequency per week in each study could affect results, additional research is necessary to verify the proper intervention period of the SFE among flatfoot individuals.

Different age groups may have divergent results. Motor performance decreases with a declined central nervous system, and altered sensory receptors, muscles, and peripheral nerves were observed in the older adults. Nevertheless, exercise interventions could lead to ameliorated motor control for this population [26]. In our included studies, all RCTs involved young adults except for one RCT which recruited middle-age adults and reported positive effects after SFE training. Consequently, the SFE could be promoted to higher age groups [18].

Considerable heterogeneity was observed among the included RCTs on account of various factors. Firstly, the progression of intervention in the SFE differed across the RCTs. Three RCT protocols progressed from a seated position to a bipedal standing position and to a unipedal standing position [18,19,20]. In the others, the progression was made in only a unipedal standing position, only a seated position, and from a seated position to a bipedal standing position [16,17,21]. Secondly, frequencies and durations of SFE sessions varied between the RCTs. Thirdly, the physical therapists’ or relevant researchers’ expertise and teaching skills may have differed from each other in spite of the fact that none of the RCTs reported this information. Fourthly, muscle hypertrophy was evaluated by different measuring instruments. Such diversity among the RCTs contributed to heterogeneity.

This study has several limitations. Firstly, several study biases may affect the evaluation of outcomes, including bias arising from the randomization process, deviations of intended interventions, and measurements of outcomes. Secondly, some RCTs had a relatively small sample size. Thirdly, the RCTs did not include participants aged above 50 years old, or less than 18 years old, and hence, a conservative attitude toward our result should be kept when addressing children or seniors. Fourthly, the long-term effects of the SFE are still uncertain. Thus, these limitations might limit the extensibility of SFE practice.

## 5. Conclusions

Our meta-analysis concluded that the SFE significantly normalized foot alignment compared with other interventions despite the fact that such a difference was not observed in muscle hypertrophy. Therefore, we recommend the SFE as a useful tool to deal with foot alignment in the flatfoot population and the concept can be incorporated into other rehabilitation or fitness programs in response to high demands on the foot. However, additional well-constructed and large-scale RCTs are necessary to determine a proper and specific protocol as well as the long-term effects of the SFE.

## Figures and Tables

**Table 1:** Characteristics of the included studies.

Author (Year)	Study Design	Inclusion Criteria	Number of Patients (Male, %)	Age, Year, Mean ± SD	BMI, kg/m^2^, Mean ± SD		Intervention
Jung (2011)	RCT	Bilateral pes planus foot with RCSP ≥4 of eversion and ND exceeding 13 mm	SG: 14 CG: 14	SG: 22.36 ± 2.41 CG: 21.93 ± 2.73	Height SG:164.89 ± 8.82 CG: 167.96 ± 7.27	Weight SG: 57.71 ± 9.65 CG: 58.93 ± 7.84	SG: Foot orthoses and short-foot exercise twice daily for 8 weeks CG: Foot orthoses for 8 weeks
Kim (2016)	RCT	Subjects whose dominant foot had flexible flatfoot according to ND exceeding 10 mm	SG: 7 (85.7) CG: 7 (57.1)	SG: 24.0 ± 1.9 CG: 24.1 ± 1.5	Height SG: 172.2 ± 6.9 CG: 167.0 ± 6.7	Weight SG: 68.2 ± 12.9 CG: 63.3 ± 17.6	SG: Short-foot exercise 30 min at a time, three times per week for 5 weeks CG: Insoles in the shoes and walk 30 min at a time, three times per week for 5 weeks
Kısacık (2021)	RCT	Patellofemoral pain patients with pronated foot defined by FPI-6 score ≥ 6	SG: 15 CG: 15	SG: 39.60 ± 8.87 CG: 43.60 ± 7.76	SG: 25.36 ± 5.19 CG: 25.09 ± 3.77		SG: Supervised hip and knee strengthening and stretching exercises, and short-foot exercise 2 times per week for 6 weeks CG: Supervised hip and knee strengthening and stretching exercises 2 times per week for 6 weeks
Okamura (2020)	RCT	Participants with FPI-6 score ≥ 6	SG: 10 (10) CG: 10 (20)	SG: 19.7 ± 0.9 CG: 20.2 ± 1.5	SG: 19.8 ± 1.4 CG: 21.1 ± 2.1		SG: Short-foot exercise three times per week for 8 weeks CG: No intervention
Pabón-Carrasco (2020)	RCT	FPI-6 score ≥ 6 in both feet to identify pronator individuals	SG: 42 (57.1) CG: 43 (41.9)	SG: 19.45 ± 0.38 CG: 20.92 ± 1.1	SG: 24.13 ± 4.16 CG: 21.65 ± 3.35		SG: Short-foot exercise daily for 4 weeks CG: Dorsal and plantar flexion exercise of the metatarsophalangeal joints daily for 4 weeks
Park (2021)	RCT	BMI ≥ 25 kg/m^2^, ND ≥ 10 mm, and inner longitudinal arch angle ≥ 150	SG: 12 (58.3) CG: 12 (58.3)	SG: 23.25 ± 1.22 CG: 24.00 ± 1.48	SG: 29.34 ± 2.81 CG: 29.39 ± 4.57		SG: Short-foot exercise 3 times a week for 20 min over 4 weeks CG: Proprioceptive neuromuscular facilitation 3 times a week for 20 min over 4 weeks

Abbreviations: SG, short-foot exercise group; CG, control group; RCT, randomized controlled trial; RCSP, resting calcaneal stance position; FPI-6, foot posture index; ND, navicular drop.

**Table 2:** Methodological quality assessment of included studies.

Author (Year)	Bias Arising from Randomization Process	Bias Due to Deviations from Intended Interventions	Bias Due to Missing Outcome Data	Bias in Measurement of the Outcome	Bias in Selection of the Reported Result	Overall Risk of Bias
Jung (2011)	Some concerns ^a^	Some concerns ^c^	Low risk	Low risk	Low risk	Some concerns
Kim (2016)	Some concerns ^a^	Some concerns ^c^	Low risk	Some concerns ^d^	Low risk	High risk
Kısacık (2021)	Low risk	Some concerns ^c^	Low risk	Some concerns ^d^	Low risk	Some concerns
Okamura (2020)	Some concerns ^a^	Some concerns ^c^	Low risk	Low risk	Some concerns ^f^	High risk
Pabón-Carrasco (2020)	Some concerns ^b^	Low risk	Low risk	Low risk	Low risk	Some concerns
Park (2021)	Some concerns ^a^	Some concerns ^c^	Low risk	Some concerns ^e^	Low risk	High risk

Methodological quality assessment was based on the Cochrane risk of bias tool (RoB2.0). ^a^ No information about concealment of the allocation sequence. ^b^ Baseline differences between intervention groups was found, but this would probably not suggest a problem with the randomization process. ^c^ Participant and/or personnel were aware of intervention groups and unclear information of non-protocol interventions. ^d^ Lack of information on outcome assessors being aware of intervention received by participants. ^e^ Outcome assessors were aware of the intervention received and the assessment of the outcome and could have been influenced by this knowledge. ^f^ Outcome data reported as median and interquartile range.

## Data Availability

The data that support the findings of this study are available from the corresponding author upon reasonable request.
